# GWAS identifies novel loci linked to seedling growth traits in highly diverse barley population under drought stress

**DOI:** 10.1038/s41598-025-94175-y

**Published:** 2025-03-24

**Authors:** Abdelfattah Badr, Hanaa H. El-Shazly, Mayada Mahdy, Matías Schierenbeck, Radwa Y Helmi, Andreas Börner, Helmy M Youssef

**Affiliations:** 1 Botany and Microbiology Department, Faculty of Science, Helwan University, Ain Helwan, Cairo, 11790 Egypt; 2 Biological and Geological Sciences Department, Faculty of Education, Ain Shams University, Cairo, 11341 Egypt; 3 Physiology and Cell Biology Department, Leibniz Institute of Plant Genetics and Crop Plant Research (IPK), Corrensstr. 3, OT Gatersleben, D-06466 Seeland, Germany; 4 Genebank Department, Leibniz Institute of Plant Genetics and Crop Plant Research (IPK), Corrensstr. 3, OT Gatersleben, D-06466 Seeland, Germany; 5 Genetics and Cytology Department, Biotechnology Research Institute, National Research Centre, Dokki, Giza, 12622 Egypt; 6 Faculty of Agriculture, Cairo University, Giza, 12613 Egypt; 7 Institute of Agricultural and Nutritional Sciences, Chair of Plant Breeding, Martin Luther University Halle-Wittenberg, Betty-Heimann-Str. 3, D-06120 Halle (Saale), Germany

**Keywords:** Barley, GWAS, PEG, Drought stress, Drought tolerance index, Climate change, Developmental biology, Plant sciences

## Abstract

Climate changes refer to long-term shifts in temperature and weather patterns that may cause drought, one of the major stresses hindering seed germination, plant growth, and crop productivity. Barley (*Hordeum vulgare* L) is considered one of the most drought-stress-tolerant cereals and may be used for elucidating genes for drought tolerance at seed germination and seedling stages that would pave the way toward improving the performance of all cereals. The current study was performed at IPK-Gatersleben (Germany) in 2023. Our aim was to explore the genetic basis of germination and seedling traits under drought stress (20% PEG_6000_ treatment) in a 198 global spring barley collection genotyped with 38,632 SNPs via Genotyping by Sequencing (GBS). The drought treatment significantly reduced the seed germination parameters and seedling traits in the genotypes of a global barley collection. Drought tolerance indices (DTI) for the measured germination and seedling traits indicate delayed and lower germination speed under drought stress than the control. The shoot fresh weight was the most affected trait, with a DTI of 37.4, followed by the seedling fresh weight SDLFW (DTI = 46.3) and root fresh weight (DTI = 47). In contrast, the root length DTI was the least affected trait by drought (78.2), followed by RDW DTI (72.8). GWAS was conducted using single-locus (CMLM) and multi-locus models (MLMM, Farm-CPU, BLINK), with significant marker-trait associations determined at -log10 (1.29E^-06^) ≥ 5.88. In our present study, we identified 79 highly significant SNPs distributed across the seven barley chromosomes related to the germination and seedling growth parameters under both control and drought conditions. Gene annotation of these highly significant SNPs revealed that 35 SNPs were in the exonic regions of genes that play roles in important plant biological and physiological processes. Further analysis exhibited 35 high-confidence candidate genes influencing barley germination and seedling growth under control and drought conditions. These genes represent promising targets for breeding and genetic enhancement efforts to improve drought tolerance in barley, potentially extending these benefits to other cereal crops.

**Supplementary Information:**

The online version contains supplementary material available at 10.1038/s41598-025-94175-y.

## Introduction

The predicted global climatic change shall reduce the productivity of most crops and induce a detrimental impact on the ecological fitness of cultivated plants^1^. The Food and Agriculture Organization (FAO) Strategy on Climate Change 2022–2031 warned about the risk factors for agriculture productivity through changes in rainfall distribution, loss of soil fertility and organic carbon depletion, increased evaporation and transpiration, increased soil salinity, pollution and increased pests and diseases^2^. One important approach to minimize the impact of climate change on food production is breeding drought-resilient water-conserving crops through genetic improvement of crop’s tolerance to environmental change. Genes for resilience environment extremes are to be found in plant genetic resources (PGRs) of crops germplasm, such as collections of landraces and crop wild relatives, which are often conserved in gene banks. The PGRs have been recognized as indispensable sources of genotypic variation required for the future breeding of new crop varieties^3^. In the recent few decades, huge efforts have been made to analyze crops’ genetic resources for material that would be adapted to climate change for updating the global plan of action for the conservation and sustainable utilization of PGR^4^.

Genome-wide association analysis (GWAS) was applied as a powerful new-generation sequencing tool to reveal the complex pathways of abiotic stress tolerance in barley^5^. Meanwhile, Cockram et al. introduced the GWAS to barley genome and mapped candidate polymorphism using 32 phenotypes and 15 morphological traits across 500 cultivars genotyped with 1,536 single nucleotide polymorphism (SNPs)^6^. Wang et al. (2012) reported significant associations for 16 morphological and nine agronomic traits in a collection of 615 barley cultivars genotyped by GWAS analysis^7^. GWAS has been successfully applied as a promising approach to defining the causative allele(s)/loci that can be used in breeding crops for adaptation to climate change. The approach for basic genetic and statistical concepts of GWAS and explained how the candidate gene(s) for specific traits can be detected using bioinformatic tools was described by Sayed et al.^8^.

Recently, the GWAS analysis has been widely applied in barley. Geng et al. (2021) identified 14 stable marker-trait associations (MTAs) for grain β-glucan content and confirmed a significantly positive correlation between grain β-glucan content and the number of favorable alleles of 14 stable MTAs. One of the putative genes, *HORVU6Hr1G088380*, could be an important gene controlling barely β-glucan content, with the SNPs being closely linked in all tested accessions and divided into two haplotypes^9^. Recently, GWAS analysis was performed for water content (WC) in 100 barley accessions grown under drought stress in a greenhouse and a growth chamber experiment. This study reported that shoot water content showed a distinct difference among barley genotypes under water stress and identified 20 significant SNPs with 41 genes associated with water stress tolerance^10^. The study by Teklemariam et al. on Ethiopian barley terminal drought stress tolerance under field and climate chamber conditions proved reduced grain biomass by 47% and 80% under field and controlled conditions, respectively, and a significant reduction in the days to maturity and plant height, in both experiments^11^.

Germination and the seedling stage of cereals are sensitive to drought stress and can be used for screening germplasm for drought tolerance. This view has been confirmed by studies on maize^12–15^, wheat^16–18^ and barley^19^. These studies agree on a similar set of drought-responsive traits, including germination percentage (G%) and germination speed (G-speed), and measurements and calculations of the shoot and root length and fresh and dry biomass. These findings encourage a consensus that germplasm screening for climate change resilience genotypes may be performed at the germination and seedlings stage by valid differences of selected traits under normal conditions and simulated drought stress imposed by 20% PEG_6000_ treatments^15^. In barley, drought tolerance during seed germination and seedling establishment is under polygenic control. Interestingly, drought tolerance in barley is consistent when determined at the three important growth stages: the germination and seedlings stage, the vegetative growth stage, and the flowering and yield stages^20^. Abdel-Ghani et al. and Hanafy et al. identified the seven root-related genes, *HERK2*, *HvARF04*, *HvEXPB1*, *PIN5*, *PIN7*, *PME5*, and *WOX5* as promising candidates for root and shoot architecture traits at seedling stage^21,22^.

Barley (*Hordeum vulgare* L.) is an ancient annual cereal. Genetic and archaeological evidence indicated that domesticated barley was a mosaic crop developed from different populations in its domestication area in the Middle East^23–25^. Barley is the fourth cereal in terms of crop production and cultivated area worldwide; it has multipurpose uses as human food, animal feed and in the brewing, industry and premeditated as an exemplary plant for temperate cereals^26^. The statistics of the worldwide production of grains in 2023/24, indicated that barley production came to about 142.87 million metric tons^27^. Barley germplasm grows in a range of climates around the world than any other cereal and can grow and ripen in a shorter time than any other cereal to the extent that Gürel et al. recommended barley genes as tools to confer abiotic stress tolerance in other crops^28^. Elakhdar et al. confirmed that drought stress on barley is a complex challenge due to the involvement of many genes, including redundant regulatory genes that control several morphological and physiological responses to drought at different stages of plant growth^29^.

The genetic diversity in barley germplasm could be further exploited to identify stress-resilient genotypes and genes^30^. Moursi et al. identified 71 quantitative trait loci (QTLs), associated with drought tolerance at seed germination and seedling stages in 60 barley genotypes, distributed across the seven chromosomes of barley, including 33 QTLs for root-length-related traits and hotspots of QTLs for various other traits^31^. Gene annotation analysis revealed candidate genes that can be targeted to select for drought tolerance. Sayed et al. using GWAS analysis, identified 38 highly significantly associated SNP markers under control and/or salinity stress. Two of the SNP markers on chromosome (chr) 1 H, two on chr 3 H, and one on chr 4 H were significantly linked to seedling fresh and dry weight under salinity stress treatment. In addition, two SNP markers on chr 7 H were also significantly associated with seedling fresh and dry weight under control conditions^8^. Khodaeiaminjan et al. identified 52 QTL by at least two GWAS approaches representing promising candidate genes with a role in root development and adaptation to drought stress in two-row spring barley landraces in well-watered and osmotic stress conditions^32^.

The Gene Bank Department of the IPK hosts most of the cereals and medicinal plant’s genetic resources collections in Germany and is one of the largest gene banks in the World, including the materials used to uncover the molecular basis of drought tolerance during seed germination and seedling establishment allows the development of stress-tolerant genotypes^19,21^. Uniformity of germination under stressful environments is vital for crop establishment and subsequent growth and yield productivity. Identification of genes involved in controlling seed germination and seedling growth under drought stress may be regarded as a prerequisite for further increasing yield potential. We aimed in this study at the identification of drought-responsive QTLs and subsequently genes that might play a role in the regulation of germination and seedling growth traits in a large and diverse global collection of barley genotypes from the IPK-barley global collection.

## Materials and methods

### Seed material and experimental design

Seed material representing 198 barley genotypes (*Hordeum vulgare* L.) was used in this study and were from the IPK Gene Bank collection based on their worldwide diversity (origin, row types, biological status, Table S1). Seeds of the control and drought treatments were germinated according to the International Seed Testing Association (ISTA) protocol (2014). A total of 300 healthy seeds (grains) of each genotype were used for the control and control treatments (150 grains each). The 150 seeds of each treatment were divided into three sets of 50 seeds. Each 50 seeds were weighted and germinated in special blotting paper sheets (Ahlstrom Munnksjö, 25 cm high × 60 cm wide) moistened either with distilled water for the control treatment or with 20% *m/v*, PEG_6000_ for simulated drought stress. Then, the sheets were rolled to separate seeds from each other and held in transparent plastic bags and incubated in a growth cabinet (ASECOS EN 1440-2) at 25 ± 2/20°C ± 2 (day/night) at a relative humidity of 60% under 16 h light/8 h dark at a light density of 400 µmol photons m^− 2^s^− 1^. Preliminary tests using 10%, 15%, and 20% *m/v*, PEG_6000_ confirmed that the 20% *m/v*, PEG_6000_ concentration is the appropriate drought stress that can illustrate the impact of drought stress on a diverse collection of barley accessions.

### Evaluation of germination percentage and recording seedling traits

Evaluation of germination percentage (G%) was made every two days from seeds sowing for the control and drought treatments for ten days. Seeds that had a minimum radicle length of 2 mm were counted as germinated. The moisture of the blotting paper rolls was continuously monitored for both treatments by adding H_2_0 or 20% PEG6000 solution as required. The number of germinated seeds for each set was counted every two days for ten days and the germination percentage for the control and drought stress treatments was calculated after ten days. Germination parameters were assessed according to the ISTA rules and seedlings that grew into healthy seedlings after 16 days of germination were used for fresh weight (FW) and shoot and root length measurements. Three plants of each replicate were used for the shoot and root FW measurements and measured parts were dried at 70 ± 5 °C for 48 h, then their dry weight (DW) was determined. The germination and seedling traits description and abbreviations under the control condition and the drought stress treatment are given in Table S2.

### Data analyses

The mean and standard deviation (SD) of each trait for each genotype under control and PEG stress treatments were determined using GenStat Release^33^. Box and Whisker charts were plotted with SRPLOT^34^ to illustrate the variation of the G% every two days for ten days of sowing for germination under the control condition and the 20% PEG_6000_ treatment (G%nd = G% after n days, and G-Speed = Germination speed). Box and Whisker charts were also performed after 16 days of sowing for eight seedling traits and seedlings exposed to the 20% PEG_6000_ treatment as described above. The ratio of the G% of seeds exposed to the 20% PEG_6000_ treatment to the germination percentage of the control seeds was calculated for all genotypes. Similar indices expressing the magnitude of change in the seedling fresh weight (SDLFW), shoot length (SHL), shoot fresh weight (SHFW), root length (RL), root fresh weight (RFW), and root dry weight (RDW), for the control plants and plants exposed to 20% PEG_6000_ using Excel 2016 (Table S3).

### ANOVA analysis of phenotypic data

Analysis of variance (ANOVA) was conducted to compare genotypes and traits using GENSTAT for Windows Ver. 19 (VSN International, Hemel Hempstead, UK) for the germination and the seedling shoot and root traits. The probability of significance in ANOVA (*P* ≤ 0.05) was used to indicate significant differences among genotypes (G), treatments (T), and interaction effects (T ^x^ G). Means were separated according to Fisher’s Least Significant Difference (LSD) at 0.05 levels of probability. GenStat 19 software was also used for broad-sense heritability (*H*^*2*^) calculations, where the mean squares for accessions (σg2), genotype × environment interaction (σgy2), and residual error (σe2), and y represents years and r (replicates). MVApp v2.0^35^ was used for correlation boxplot calculations. The restricted maximum likelihood (REML) algorithm was applied and the Best Linear Unbiased Estimators (BLUEs) for each treatment were calculated (BLUE-C and BLUE-S) using the nlme package in R^36^.

The response of the measured G% for ten days, G-speed, and seedling traits to drought as a percentage of their corresponding control was calculated as the Drought Tolerance Index (DTI) for all barley genotypes. The grand average of the DTIs of all genotypes was calculated and used as a measure for drought tolerance of the examined barley collection to evaluate these traits’ response to the drought stress treatment compared to the control (Table S3).

### Genotyping of the barley panel

Genotypic information for the 198 accessions yielded a total of 38,632 SNPs. This dataset was assembled by SNPs from the genotyping by sequencing technique (GBS). All used SNPs were subjected to a quality check where Minor Allelic Frequency (MAF) ≥ 1% and 80% presence rate^37–39^.

### Genome-wide association study (GWAS) and candidate genes detection

Different GWAS models comprising single-locus, such as the Compressed Mixed Linear Model (CMLM)^40^, Multi-locus methods like Multiple Loci Mixed Model (MLMM)^41^, Fixed and random model Circulating Probability Unification (Farm-CPU)^42^ and the Bayesian information and Linkage-disequilibrium Iteratively Nested Keyway (BLINK)^43^ were tested to detect reliable marker-traits association using the Genomic Association and Prediction Integrated Tool (GAPIT 3 version updated April 2023)^44^ in the R 4.4.0 “Puppy Cup”. The detected associations above the threshold suggested in GAPIT3 (*P* = 0.05/n, n = total number of SNP used) equal to -log10 (1.29E-^06^) ≥ 5.88 were considered significant marker-trait associations. The effect of the significant markers and phenotypic variance explained by the associated markers (PVE) was taken out from the GWAS analysis output.

The physical position of QTNs and candidate genes were defined using the barley database BARLEX of Morex version 3^38^. The Barley database was used for molecular and cellular characterization and gene annotations of the candidate genes (https://apex.ipk-gatersleben.de/apex/f?p=284:10::).

## Results

### Variation in germination percentage and germination speed

The average of 50 seed weights for all barley genotypes showed non-significant differences, confirming the unbiased selection of grains for investigating germination and early seedling growth under drought stress compared to normal conditions (Table 1). The G% evolution (G%2d, G%4d, G%6d, G%8d, and G%10d) and G-Speed were calculated as the mean, minimum, maximum; Variance, coefficient of variation, SD: standard deviation (Table 1). A comparison of the G% evolution indicated substantial variation under control conditions and simulated drought stress by the 20% PEG_6000_ treatment.

**Table 1:** Summary statistics of G%, G-SP and seedling traits of a global collection of 198 barley genotypes evaluated under control (C) and drought stress (D) tolerance at the seedling stage.

Traits abbreviation	Treatment	Mean	Min.	Max.	Var.	Median	%CV	SD
50 Seed wt. (g)	C	2.779	1.68	3.62	0.131	2.78	13.02	0.362
	D	2.719	1.58	4.50	0.168	2.74	15.06	0.409
G%2d	C	78.95	0	100	333.6	84	23.13	18.26
	D	7.05	0	40	41.69	6	91.53	6.46
G%4d	C	90.85	4	100	162.2	95	14.02	12.74
	D	65.12	0	100	448.0	70	32.50	21.17
G%6d	C	92.70	16	100	107.1	96	11.17	10.35
	D	81.69	0	100	293.8	86	20.98	17.08
G%8d	C	93.18	16	100	96.22	96	10.53	9.81
	D	85.59	8	100	217.0	90	17.21	14.73
(G10d%)	C	93.18	16	100	96.22	96	10.53	9.81
	D	85.74	8	100	216.5	90	17.17	14.71
G-SP	C	49.30	3.63	57.08	58.70	51.44	15.54	7.66
	D	26.35	0.9	41.58	39.00	27.64	23.70	6.24
SDLFW (g)	C	0.54	0.3	0.85	0.0062	0.54	14.57	0.079
	D	0.25	0.09	0.50	0.0024	0.25	19.57	0.049
SHL (cm)	C	25.02	14.00	33.00	8.98	25.00	11.98	2.99
	D	13.29	3.50	24.00	8.96	13.00	22.52	2.99
SHFW (g)	C	0.289	0.16	0.59	0.0028	0.28	18.40	0.0533
	D	0.108	0.03	0.60	0.00117	0.10	31.75	0.0343
SHDW (g)	C	0.0215	0.01	0.0383	0.000015	0.0212	18.27	0.0039
	D	0.0131	0.005	0.0283	0.000008	0.013	22.21	0.0029
RL (cm)	C	14.61	7.5	24	5.09	14.5	15.45	2.26
	D	11.43	5.5	18	3.63	12	16.67	1.90
RFW (g)	C	0.168	0.08	0.33	0.0013	0.17	21.66	0.036
	D	0.079	0.02	0.14	0.0004	0.07	29.18	0.021
RDW (g)	C	0.0125	0.0055	0.063	0.000013	0.0123	29.16	0.00365
	D	0.0091	0.0018	0.098	0.000018	0.0095	43.90	0.00435

For genotypes passport data and detailed results, see data in Supplementary Table 1. Seed wt. = Seed weight, Germination % = G%, G%2d = G% after 2 days, G4d% = G% after 4 days, G6d% = G% after 6 days, G8d% = G% after 8 days, G10d% = G% after 10 days.

*Min.* Minimun, *Max.* Maximun, *Var.* Variance, *CV* coefficient of variation, *s.d* standard deviation, *G-SP* germination speed, *SDlFwtW* seedling’s fresh weight, *SHL* shoot length, *SHFW* shoot fresh weight, *RL* root length, *RFW* root fresh weight, *RDW* Root dry weight.

**Fig. 1:**
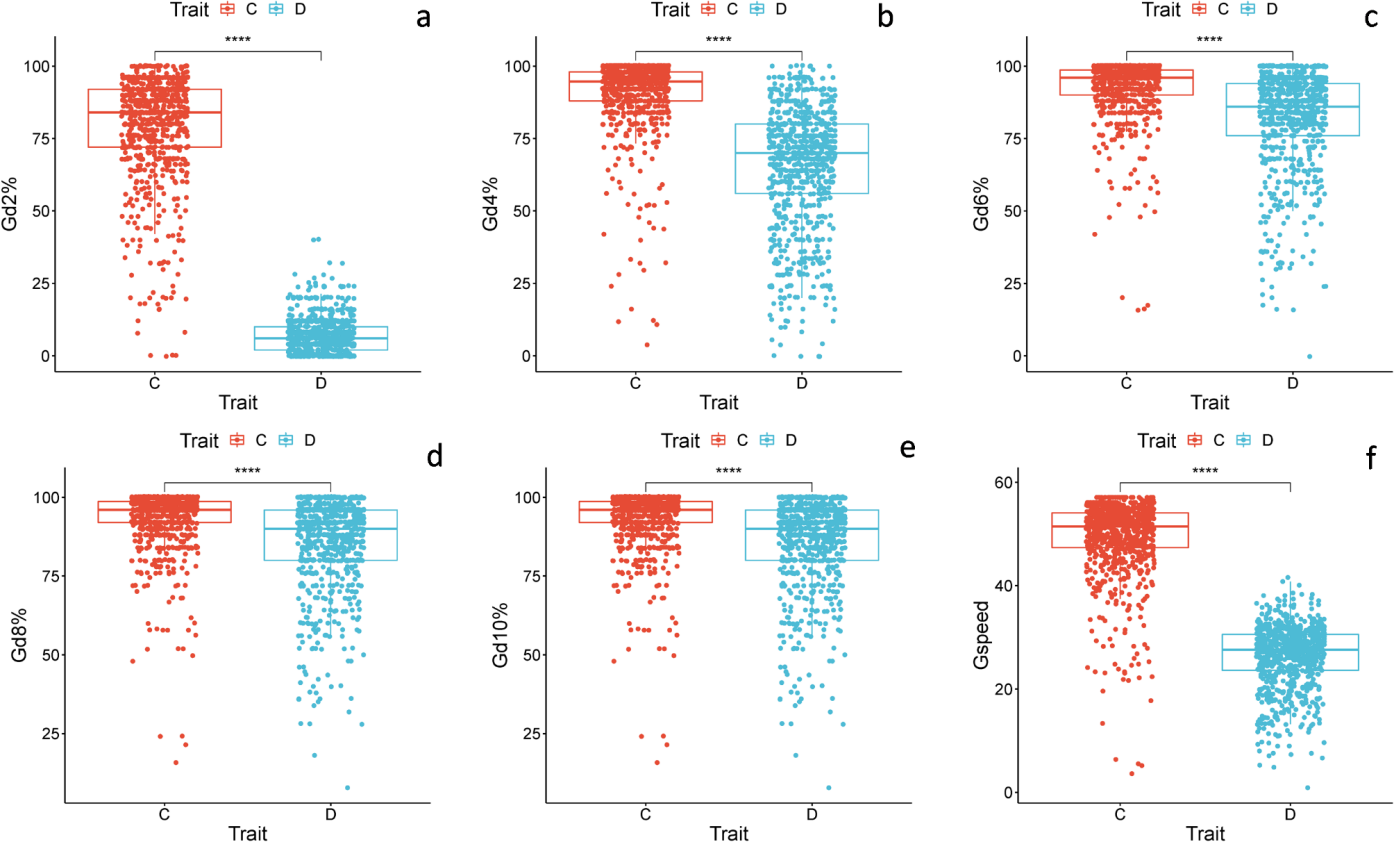
(**a**–**f**) Boxplots for germination traits in 198 barley genotypes under control and drought stress conditions (PEG 20%). (**a**) Germination percentage after 2 days; (**b**) Germination percentage after 4 days; (**c**) Germination percentage after 6 days; (**d**) Germination percentage after 8 days; (**e**) Germination percentage after 10 days; (**f**) Germination speed. Significance level: *p < 0.05; **p < 0.01, ***p < 0.001; ****p < 0.0001.

Box and Whisker charts for (G%) every two days and germination speed, which reflects variation among genotypes, are illustrated in Fig. 1(a-f). Substantial variations are evident among the genotypes, as indicated by the abundance of outlier genotypes beyond the lower and upper limits of the boxplots for the G%.

The G% of all accessions varied significantly, as indicated by the ANOVA analysis under both control and the 20% PEG_6000−_induced drought stress (Table 2). After two days of germination under control conditions, the mean G%2d was 78.95%. Whereas under drought stress, the mean G%2d showed initial lagging in the onset of germination, as indicated in Fig. 1a (7.05%). As time proceeded, the G%4d under the PEG_6000_ stress increased to a mean of 65.12% but remained much lower than the control mean value of 90.39%. The G%6d slightly increased to 92.70% under control conditions, but under the 20% PEG_6000_ drought stress, the G%6d increased further to 81.69%. However, the G%8d scored a slight and final increase to 93.18% under normal conditions compared to 85.59% under drought. No further increase in G%10d under the control or drought conditions (Table 1). The G-speed was highly significantly increased under the control treatment (Mean = 49.30) than the G-speed under drought stress treatment (Mean = 26.35).

**Table 2:** Means square and p-value (ANOVA) of barley seed G% for ten days under normal and simulated drought stress imposed by 20% PEG_6000_ concentration, G-SP, and seedling growth traits in an experiment with a global collection of 198 barley genotypes.

Traits	Treatment (T)	Genotype (G)	T × G	H^2^ C/D
Seed (wt)	1.10**	0.792**	0.044**	0.984/0.980
(G%2d)	1,535,187**	576.9**	414.5**	0.969/0.930
(G%4d)	196,634**	1180.2**	373.1**	0.972/0.948
(G%6d)	35,981**	819.4**	199.2**	0.969/0.948
(G%8d)	17,076**	636.0**	138.5**	0.968/0.934
(G%10d)	16,594**	633.4**	139.96**	0.968/0.934
(G-SP)	156,500**	215.1**	46.72**	0.977/0.957
SDlFW	24.61**	0.013**	0.0058**	0.893/ 0.921
SHL	40,830**	29.86**	16.42**	0.950/0.966
SHFW	9.77**	0.0046**	0.0034**	0.897/0.813
SHDW	2.11e^− 2^**	3.40e^− 5^**	1.83e^− 5^**	0.891/0.930
RL	3006.7**	11.64**	4.62**	0.814/0.886
RFW	2.824**	0.0025**	0.0010**	0.868/0.850
RDW	0.002**	0.00003**	0.000018**	0.805/0.705

For genotypes passport data and detailed results, see Supplementary Table 1.

*H*^*2*^ broad-sense heritability.

**indicate significance *P* < 0.001; *P < 0.05; ns (no significative).

*Seed wt* seed weight, *G%2d* germination% after 2 days, *G%4d* germination% after 4 days, *G%6d* germination% after 6 days, *G%8d* germination% after 8 days, *G%10d* germination% after 10 days, *G-SP* germination speed, *SDLFW* Seedling’s fresh weight, *SHL* shoot length, *SHFW* shoot fresh weight, *RL* root length, *RFW* root fresh weight, RDW root dry weight.

**Fig. 2:**
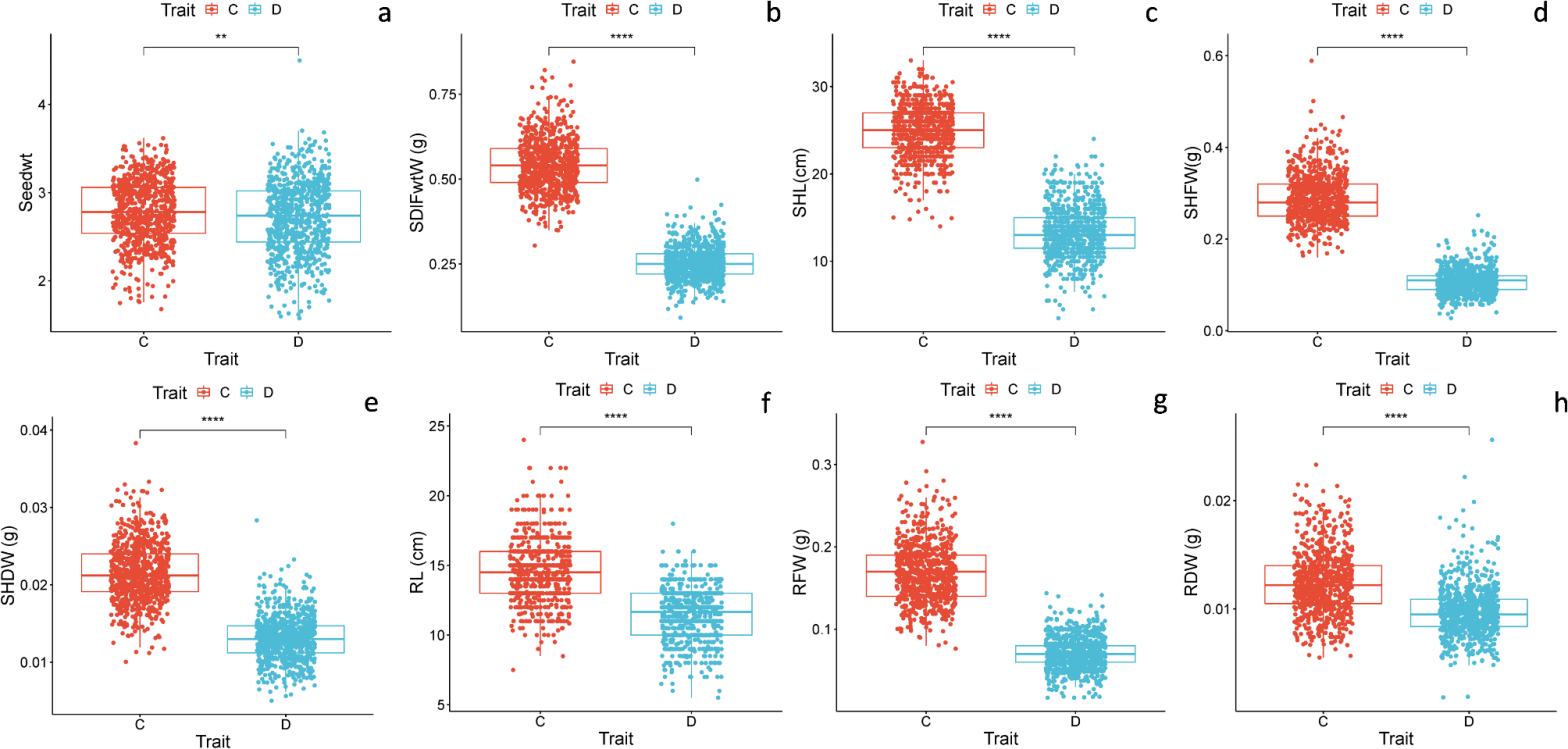
(**a**–**h**). Boxplots of seedling growth parameters in 198 barley genotypes under control and drought stress conditions (PEG 20%). (**a**) Seed weight (**b**) Seedling fresh weight (**c**) Shoot length (**d**) Shoot fresh weight; (**e**) Shoot dry weight; (**f**) Root weight; (**g**) Root fresh weight; (**h**) Root dry weight. Significance level: *p < 0.05; **p < 0.01, ***p < 0.001; ****p < 0.0001.

Means square and p-value (ANOVA) of barley seed germination and seedling growth traits, taking into consideration the 198 barley genotypes, indicate significance *P* < 0.001 for treatment (T) and genotype (G) and T^x^G. The figures illustrating the Box and Whisker charts for germination under control and drought stress conditions are shown in Fig. 2(a-h). They show that the G%2d under control showed extreme variation among genotypes as indicated by the scattering of outliers of the genotype above and below the boxplots (Fig. 1a). In the same figure, the delay in the onset of germination of all genotypes under drought stress showed little variation among genotypes at this early stage of germination. The G%2d (Fig. 1b) showed extreme variation among genotypes compared to the control. The difference among genotypes in the G% continued to be more evident after six days and eight days of germination (Fig. 1c, d). The variation among genotypes in G-speed is also indicated by the upper and lower values of G-speed as illustrated in Fig. 1f.

### Variation in seedling’s growth traits

The drought stress simulated by the 20% PEG_6000_ treatment retarded the seedling growth of all barley genotypes. A photograph illustrating the impact of drought stress on the seedling growth of 12 genotypes of barley after 16 days of germination is illustrated in Figure S1. Box and Whisker charts showing the variation in seedling traits for all genotypes, measured for the control seedlings and seedlings exposed to 20% PEG_6000_ treatment, are illustrated in Fig. 2(a-h). Substantial variations are evident in the measured seedling traits as indicated by the outlier’s genotypes above and below the Box Plots in all seedling traits. Summary statistics of the seedling traits of the global collection of 198 barley genotypes evaluated for drought stress tolerance are given in Table 1. The mean value of the measured traits is much higher under normal conditions compared to drought stress. For example, the mean SDLFW (0.54 g) under normal conditions is much higher than SDLFW under drought stress (0.25 g). Meanwhile, the mean shoot length (25.02 cm) under normal conditions is much higher than the SHL (13.29 cm) under drought. However, root length was less affected by drought stress than seedling fresh weight shoot weight and shoot. The mean RL was less affected by drought compared to seedling fresh weight and shoot length (14.61 cm under normal conditions vs. 11.43 cm under drought).

**Fig. 3:**
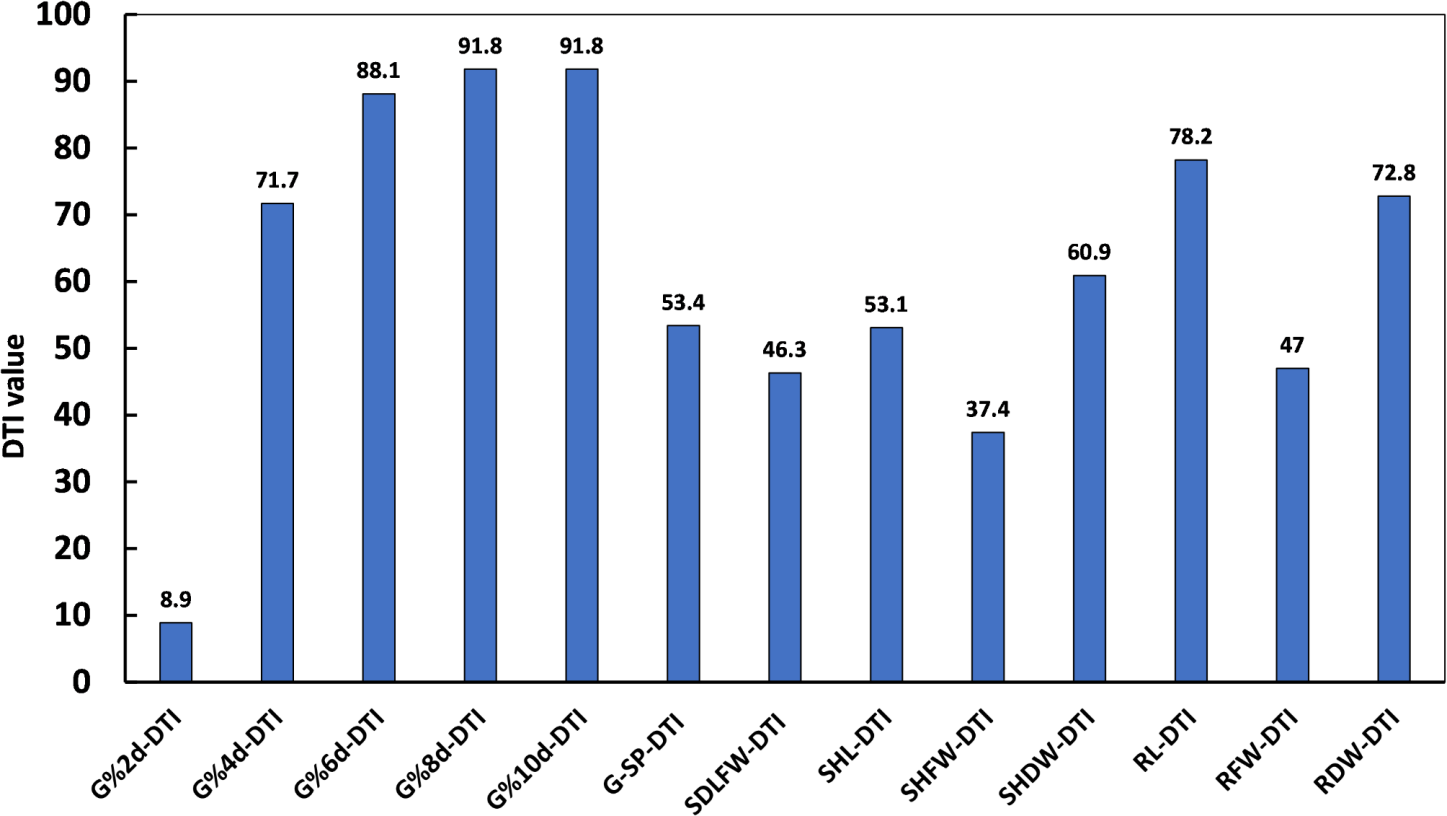
Histogram illustrating the impact of simulated drought imposed by 20% PEG on germination rate, germination speed and the measured seedling traits under drought expressed as DTI calculated as a percentage of their values under the control conditions for all barley genotypes.

Means square and p-value (ANOVA) of barley seedling growth traits,, also indicated significance *P* < 0.001 for treatment (T) and genotype (G) and T^x^G. Figure 3 confirms the similar average weight for grains for all barley genotypes used in the current study (Table 1).

The calculated DTIs to evaluate traits response to the drought stress treatment compared to the control are given in Table S3. A histogram illustrating the response of the measured G% for ten days, G-speed, and seedling traits to drought as a percentage of their value under the control conditions calculated as drought tolerance index (DTI) is presented in Fig. 3. The G%2d% DTI was only 8.9%, but the G%4d DTI increased to 71.7%, and the G%6d DTI was further increased to 88.2%. The G%8d DTI (91.8) remained constant after ten days (G%8d DTI). The G-speed DTI was 53.3%, indicating a much lower G-speed under drought stress compared to the control condition. The SHFW DTI was the most affected trait (37.4%), followed by the SDLFW DTI (46.3%) and RFWD DTI (47%). On the other hand, the RL DTI value was the least affected trait by drought stress, with a value of 78.2%, followed by RDW DTI, with a value of 72.8% (Fig. 3).

**Fig. 4:**
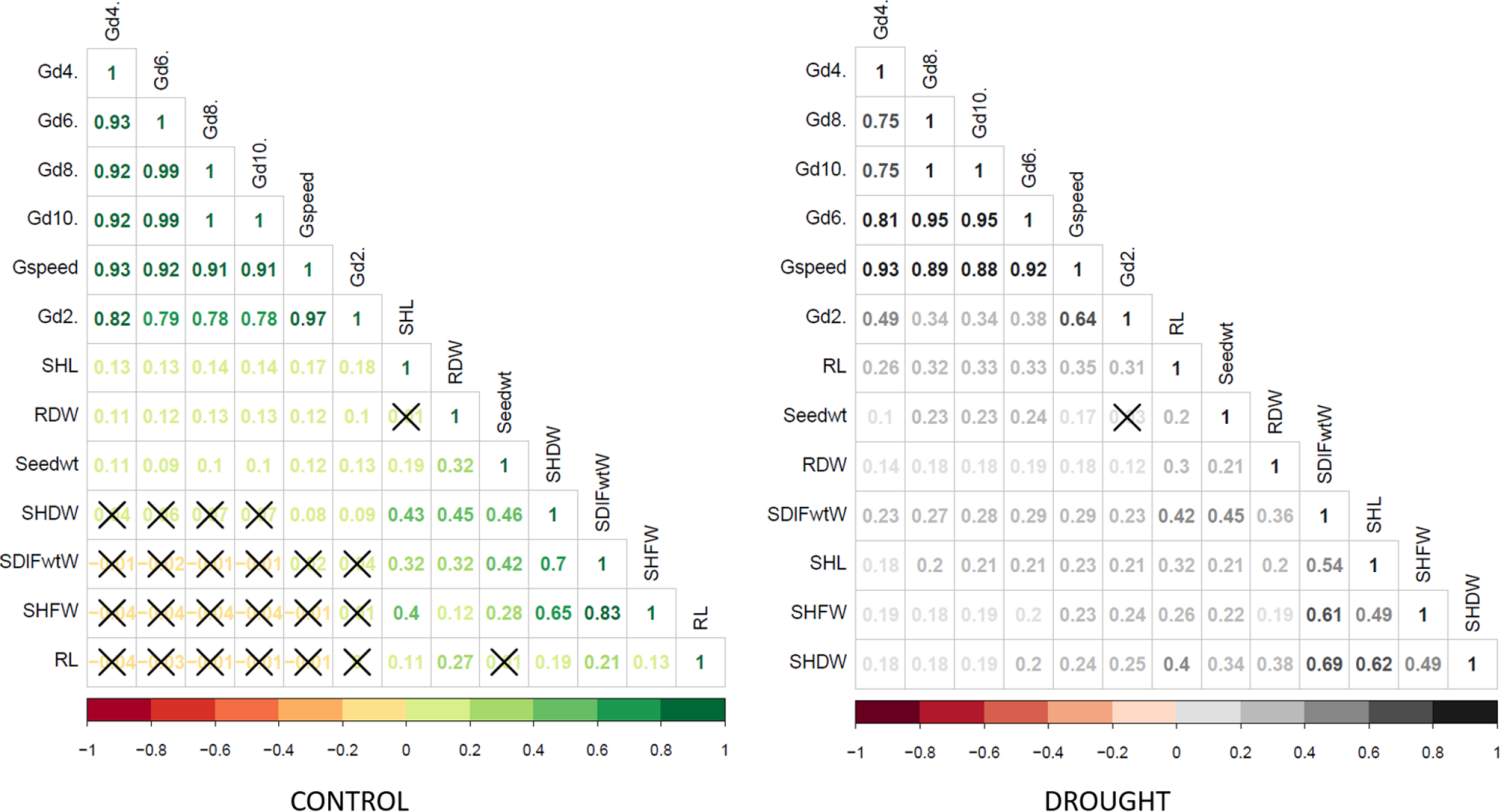
Pearson correlation coefficients of BLUEs values of the studied traits between (**A**) control and (**B**) drought (PEG20%) in 198 barley genotypes. The degree of significance for all correlations was *P* < 0.001. The color reflects the strength of the correlation. Black crosses indicate non-significant correlations.

Figure 4 illustrates the Pearson Correlation Coefficients of BLUEs values of the studied traits under the control condition (A) and the drought (B) treatments in the 198 barley genotypes. The most important correlation is found at the top of the triangle for the germination traits. Black crosses indicate non-significant correlations between. The Pearson Correlation Coefficient values for the G%2d under control are higher than the G%2d under drought stress, reflecting the initial delay of the onset of germination under drought stress. However, under the control conditions, non-significant correlations were evident between the germination traits: G%4d, G%6d, G%8d, G%10d, G-speed, and G%2d and the seedling traits; SDLFW, SHFW, and RL. Lack of correlation was also found between SHDW and G%4d, G%6d, G%8d, G%10d, and between RDW and SHL and RL and seed wt. However, positive correlations were observed for seedling traits under the control and drought stress conditions. Positive but low values of Pearson Correlation Coefficients were scored between the germination and seedling traits values under both control and drought stress conditions. However, higher correlation values were found between the germination traits G%4d, G%6d, G%8d, G%10d, and G-speed, and also between some of the seedling traits, particularly SHFW, SHDW, and SHL (Fig. 4).

QTNs and genetic regions associated with seedling growth under control and drought conditions in a global spring barley collection.

**Fig. 5:**
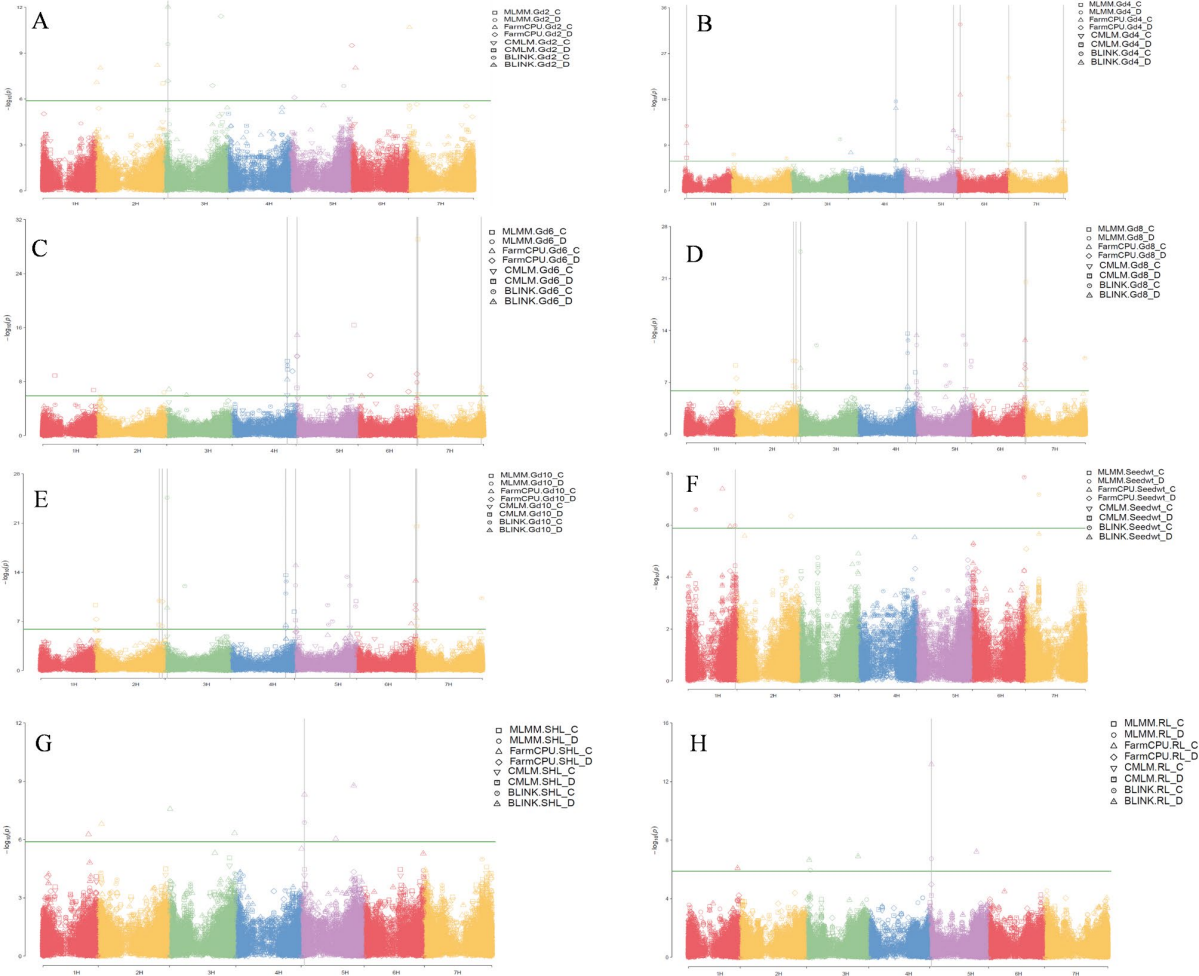
Manhattan plots generated with MLMM, Farm-CPU, CMLM, and BLINK models showing the distribution of SNPs associated with G% after 2 days (**A**), 4 days (**B**). 6 days (**C**). 8 days (**D**), germination speed (**E**) seed weight (**F**), shoot length (**G**) and root length (H) under the control conditions and drought stress conditions imposed by 20% PEG solution for the 198 barley accessions used in the current study.

Using a multi-model GWAS approach, our study identified a total of 112 QTLs with -log_10_ (1.29E-^06^) = > 5.88) distributed across the seven barley chromosomes related to diverse seedling growth parameters under both control and drought conditions (Fig. 5 and Table S4). In terms of significance, chromosomes 5 H (38), 2 H (17), 3 H (15) and 6 H (13) were found to have the highest number of QTN related to these traits, while 1 H (10), 4 H (9) and 7 H (10) exhibited a lower number of associations. Figure S2 and S3 illustrates the quantile-quantile plot for germination under control and drought stress simulated by 20% PEG_6000_, using four different GWAS methods (MLMM, Farm-CPU, CMLM, and BLINK). PCA plot for the association panel are displayed in Figure S4.

In total, 12 significant QTNs were reported for G%2d on chromosomes 2 H (4), 3 H (3), 5 H (2), 6 H (2) and 7 H (1) with a -log_10_ ranging from 6.10 to 12.03 and a phenotypic variation explained (%PVE) from 0.01 to 71.1%. About G%4d, 15 QTNs with -log_10_ ranging from 5.95 to 32.72 and %PVE from 0.01 to 78.1% were identified on 1 H (1), 2 H (2), 3 H (1), 4 H (3), 5 H (5), 6 H (1) and 7 H (2). For their part, 17 QTNs related to G%6d were reported on chromosomes 1 H (2), 2 H (1), 3 H (2), 4 H (3), 5 H (3), 6 H (3) and 7 H (3). These QTNs exhibited -log_10_ values ranging from 5.97 to 29.09 and PVE% from 0.01 to 78.7%.

Twenty-two QTNs were reported for G%8d on 2 H (4), 3 H (2), 4 H (3), 5 H (9), 6 H (2) and 7 H (2) with -log_10_ values from 6.03 to 24.62 (%PVE = 0.01 to 82.9%). On chromosomes 2 H (4), 3 H (2), 4 H (3), 5 H (10), 6 H (2) and 7 H (2) in total, 23 QTNs were detected and related to G%10d (-log_10_ 6.03–24.62; PVE% from 0.01 to 82.22%). For their part, 13 QTNs were spotted for G_speed on 1 H (2), 3 H (1), 4 H (3), 5 H (3), 6 H (2) and 7 H (2) (-log_10_ 5.89 to 14.16 and PVE% from 0.01 to 83.62%). Concerning the seedling growth parameters, 14 marker-traits associations were identified for RDW on 2 H (4 QTNs), 3 H (3), 4 H (1), 5 H (3) and 7 H (3) with -log_10_ values ranging from 5.98 to 34.77 and PVE (%) from 0.01 to 59.62%. For RL, Six QTNs were detected on 1 H (1), 3 H (3) and 5 H (2) (-log_10_ 5.95 to 13.18; PVE of 3.68 to 37.08%). In the case of SL, seven QTNs were reported on chromosomes 1 H (1), 2 H (1), 3 H (2) and 5 H (3) (-log_10_ 6.04 to 8.78 and PVE (%) ranging from 0.11 to 26.52%). Seven significant associations were reported for Seedwt on 1 H (4), 2 H, 6 H and 7 H (one each), showing a -log_10_ from 5.96 to 8.49 and PVE% ranging from 0.01 to 12.62%.

Out of the 112 significant QTLs, 42 QTNs showed a positive effect in all traits, while the other 70 significant QTLs presented a negative effect on germination and seedling growth parameters. In all of the Germination traits except the G%2d, marker (chr4H:523809780:C: A) on chromosome 4 H was presented and positively affected the germination with (6.74–15.41%), while marker (chr7H:8170605:C: G) on chromosome 7 H negatively affected all the germination traits, except G%4d, with (-10.91 to -34.77%).

More details about the QTNs detected, such as chromosome, marker position, effect, -log_10_, target and alternative allele, are displayed in Table S4.

### Candidate genes underlying barley seedling growth under control and drought stress

After reporting the most significant QTNs, a further analysis exhibited 74 high-confidence candidate genes (Table 3 and Table S5) influencing barley germination and seedling growth. In control conditions, 41 genes were detected (17 genes showing a positive effect on the traits and 24 genes had a negative effect). While under drought stress conditions, we found 33 genes affected all the traits; 10 genes had a positive effect and 23 genes had a negative effect on all traits.

**Table 3:** Candidate genes associated with positive and negative effects on germination and seedling traits under control and drought stress conditions in a global spring barley collection.

Gene HORVU ID	SNP and Position	Annotation	Chr	Traits	Allele +/−	Allele effect	
Control							
HORVU.MOREX.r3.1HG0008980.1	chr1H:21,389,172:C: G	TOX high mobility group box protein	1 H	G%4d, Gspeed	+	4.89–13.05	%
HORVU.MOREX.r3.1HG0078690.1	chr1H:482,009,779:C: T	Signal recognition particle 54 kDa	1 H	G%6d	+	0.1403	%
HORVU.MOREX.r3.1HG0054230.1	chr1H:362,456,935:A: C	MADS-box transcription factor	1 H	Seed wt	+	0.14	gm
HORVU.MOREX.r3.1HG0064790.1	chr1H:425,607,098:G: T	Microtubule associated family protein	1 H	SL	+	1.3	cm
HORVU.MOREX.r3.2HG0113120.1	chr2H:43,184,109:G: A	Pentatricopeptide repeat-containing protein	2 H	G%2d	+	0.2319	%
HORVU.MOREX.r3.4HG0395010.1	chr4H:523,809,780:C: A	Nuclear pore complex protein NUP133	4 H	G%4d, G%6d, G%8d, G%10d, Gspeed	+	6.74–15.41	%
HORVU.MOREX.r3.4HG0395710.1	chr4H:527,981,433:T: A	Caleosin	4 H	G%4d, G%8d, G%10d	+	4.74–5.27	%
HORVU.MOREX.r3.4HG0331880.1	chr4H:1,672,863:C: T	Beta-amylase	4 H	Gspeed	+	10.65	%
HORVU.MOREX.r3.5HG0426160.1	chr5H:14,470,056:G: A	Disease resistance protein (NBS-LRR class)	5 H	G%8d, G%10d	+	0.0611	%
HORVU.MOREX.r3.5HG0463050.1	chr5H:312,501,130:C: T	5’-3’ exonuclease family protein	5 H	G%8d, G%10d	+	0.0999	%
HORVU.MOREX.r3.5HG0463920.1	chr5H:320,721,391:C: G	LC	5 H	G%8d, G%10d	+	0.0448	%
HORVU.MOREX.r3.5HG0468940.1	chr5H:356,510,708:G: A	NAD(P)-binding Rossmann-fold superfamily protein	5 H	G%8d, G%10d	+	0.0311	%
HORVU.MOREX.r3.5HG0497770.1	chr5H:496,479,298:G: A	BAG family molecular chaperone regulator-like protein	5 H	G%8d, G%10d	+	0.0763	%
HORVU.MOREX.r3.5HG0448590.1	chr5H:150,482,693:T: C	WRKY transcription factor	5 H	G%4d	+	0.0505	%
HORVU.MOREX.r3.5HG0517230.1	chr5H:543,063,520:T: C	VQ motif family protein, expressed	5 H	G%4d	+	0.0441	%
HORVU.MOREX.r3.5HG0430860.1	chr5H:30,477,592:T: C	Kinase, putative	5 H	SL	+	1.03 cm	cm
HORVU.MOREX.r3.7HG0750970.1	chr7H:626,568,450:C: T	Protein DETOXIFICATION	7 H	G%8d, G%10d	+	9.22	%
HORVU.MOREX.r3.1HG0026430.1	chr1H:113,962,015:C: G	Proline-rich receptor-like kinase,	1 H	G%6d	−	−15.78	%
HORVU.MOREX.r3.1HG0022360.1	chr1H:83,116,022:A: G	transmembrane protein	1 H	Seedwt	−	−0.174	gm
HORVU.MOREX.r3.1HG0068820.1	chr1H:445,145,369:G: A	Metacaspase	1 H	Seedwt	−	−0.093	gm
HORVU.MOREX.r3.2HG0096610.1	chr2H:2,602,324:G: T	StAR-related lipid transfer protein	2 H	GH%8d, G%10d	−	−13.92	%
HORVU.MOREX.r3.2HG0192890.1	chr2H:606,315,847:T: C	Xyloglucan endotransglucosylase/hydrolase	2 H	G%4d, G%8d, G%10d	−	−8.26	%
HORVU.MOREX.r3.2HG0190170.1	chr2H:597,869,636:G: A	F-box domain containing protein	2 H	G%2d	−	−11.67	%
HORVU.MOREX.r3.2HG0107400.1	chr2H:25,328,601:A: G	Kinase family protein	2 H	G%4d	−	−9.22	%
HORVU.MOREX.r3.3HG0221170.1	chr3H:5,848,191:G: T	F-box like protein	3 H	SL	−	−1.14	cm
HORVU.MOREX.r3.3HG0325670.1	chr3H:609,276,465:G: A	F-box domain containing protein	3 H	SL	−	−1.096	cm
HORVU.MOREX.r3.3HG0228050.1	chr3H:18,765,394:A: G	E3 ubiquitin-protein ligase	3 H	G%6d, G%8d, G%10d, Gspeed	−	−5.69 /− 14.3	%
HORVU.MOREX.r3.3HG0297850.1	chr3H:531,575,910:G: A	Non-specific serine/threonine protein kinase	3 H	G%4d	−	−5.54	%
HORVU.MOREX.r3.4HG0415720.1	chr4H:602,909,318:G: A	Endoglucanase	4 H	G%8d, G%10d	−	−12.95	%
HORVU.MOREX.r3.4HG0339820.1	chr4H:27,647,432:A: G	Sugar transporter family protein	4 H	G%4d	−	−3.34	%
HORVU.MOREX.r3.5HG0464370.1	chr5H:323,072,464:T: C	Vacuolar sorting receptor protein	5 H	SL	−	−0.94	gm
HORVU.MOREX.r3.5HG0509180.1	chr5H:523,346,728:G: T	Prefoldin subunit 4	5 H	G%6d, G%8d, G%10d	−	−15.84	%
HORVU.MOREX.r3.5HG0497230.1	chr5H:494,414,973:T: C	PHD-finger family protein	5 H	G%4d	−	−10.08	%
HORVU.MOREX.r3.5HG0519230.1	chr5H:547,967,784:G: A	U3 small nucleolar RNA-associated protein 18-like protein	5 H	G%4d, G%6d, Gspeed	−	−18.06	%
HORVU.MOREX.r3.5HG0533230.1	chr5H:577,482,385:C: G	Pirin-like protein	5 H	G%8d, G%10d	−	−3.67	%
HORVU.MOREX.r3.5HG0535750.1	chr5H:582,175,268:A: T	Cleavage and polyadenylation specificity factor subunit 3	5 H	G%4d, G%8d, G%10d	−	−4.79	%
HORVU.MOREX.r3.6HG0628050.1	chr6H:548,454,590:C: T	Cytosine-specific methyltransferase	6 H	Seedwt	−	−0.175	gm
HORVU.MOREX.r3.6HG0617830.1	chr6H:518,070,091:G: A	Cyclin-dependent kinase inhibitor	6 H	G%10d	−	−11.46	%
HORVU.MOREX.r3.6HG0555330.1	chr6H:43,748,789:G: A	BURP domain protein RD22	6 H	G%2d	−	−20.42	%
HORVU.MOREX.r3.7HG0639100.4	chr7H:8,170,605:C: G	6-phosphogluconate dehydrogenase, decarboxylating	7 H	G%2d, G%4d, G%6d, G%8d, G%10d, Gspeed	−	−10.9/−34.77	%
HORVU.MOREX.r3.7HG0743140.1	chr7H:611,034,910:T: C	Transducin/WD40 repeat protein	7 H	G%4d, G%6d, GSpeed	−	−7.2/−25.87	%
Drought							
HORVU.MOREX.r3.1HG0082530.1	chr1H:490,499,372:C: A	Respiratory burst oxidase	1 H	RL	+	0.75	cm
HORVU.MOREX.r3.2HG0099620.1	chr2H:10,431,708:A: C	O-methyltransferase family protein	2 H	G%8d, G%10d	+	4.57	%
HORVU.MOREX.r3.2HG0178520.1	chr2H:544,530,240:C: G	Endoglucanase	2 H	RDW	+	0.05	gm
HORVU.MOREX.r3.2HG0205370.1	chr2H:637,063,406:T: A	Transcription elongation factor Spt5	2 H	G%6d, G%8d, G%10d	+	6.39	%
HORVU.MOREX.r3.3HG0234440.1	chr3H:36,116,907:G: C	Amino acid permease	3 H	G%2d	+	7.4	%
				RL	+	1.89	cm
HORVU.MOREX.r3.3HG0231430.1	chr3H:26,462,351:C: T	E3 Ubiquitin ligase family protein	3 H	RL	+	1.04	cm
HORVU.MOREX.r3.5HG0423130.1	chr5H:7,635,237:G: A	Thaumatin-like protein	5 H	G%6d, G%8d, G%10, Gspeed	+	8.8–26.9	%
HORVU.MOREX.r3.5HG0481010.1	chr5H:434,421,304:C: T	Initiation factor 4 F subunit	5 H	RDW	+	0.007	gm
HORVU.MOREX.r3.6HG0569310.1	chr6H:117,879,365:G: A	Plant/T31B5-30 protein	6 H	G%6d	+	9.41	%
HORVU.MOREX.r3.7HG0663860.1	chr7H:78,057,538:C: T	EF hand calcium-binding protein family	7 H	RDW	+	0.001	gm
HORVU.MOREX.r3.1HG0004220.1	chr1H:8,938,031:C: T	Cysteine proteinase	1 H	Gspeed	−	−7.0	%
HORVU.MOREX.r3.2HG0185030.1	chr2H:572,536,278:T: C	Multiprotein-bridging factor	2 H	Seedwt	−	−0.105	gm
HORVU.MOREX.r3.2HG0107400.1	chr2H:25,328,604:T: C	Kinase family protein	2 H	RDW	−	−0.013	gm
HORVU.MOREX.r3.2HG0207520.1	chr2H:641,742,088:G: A	Wound-responsive family protein	2 H	RDW	−	−0.006	gm
HORVU.MOREX.r3.2HG0189740.2	chr2H:596,332,379:G: A	Acetylglutamate kinase-like protein	2 H	RDW	−	−0.006	gm
HORVU.MOREX.r3.3HG0294370.1	chr3H:513,961,348:T: C	Methyl esterase	3 H	RL	−	−0.68	cm
HORVU.MOREX.r3.3HG0286190.1	chr3H:469,908,093:C: T	Protodermal factor 1	3 H	G%2d	−	−5.08	%
HORVU.MOREX.r3.3HG0303390.1	chr3H:552,642,883:G: A	Dirigent protein 17	3 H	G%2d	−	−4.37	%
HORVU.MOREX.r3.3HG0307390.1	chr3H:564,672,872:A: C	2-oxoglutarate (2OG) and Fe(II)-dependent oxygenase	3 H	RDW	−	−0.012	gm
HORVU.MOREX.r3.3HG0249600.1	chr3H:141,885,554:G: A	Reticulon-like protein	3 H	RDW	−	−0.006	gm
HORVU.MOREX.r3.4HG0406060.1	chr4H:572,733,912:G: T	Agmatine coumaroyltransferase-2	4 H	G%6d	−	−13.08	%
HORVU.MOREX.r3.4HG0415570.1	chr4H:602,417,124:G: A	Cortical cell-delineating protein	4 H	RDW	−	−0.001	gm
HORVU.MOREX.r3.5HG0495430.1	chr5H:490,757,115:G: A	Beta-glucosidase	5 H	SL	−	−1.33	cm
HORVU.MOREX.r3.5HG0487790.1	chr5H:467,410,034:C: T	Cyclin family protein	5 H	RL	−	−1.1	cm
HORVU.MOREX.r3.5HG0432280.1	chr5H:36,660,118:A: G	Kinase interacting (KIP1-like) family protein	5 H	G%2d	−	−2.52	%
HORVU.MOREX.r3.5HG0505720.1	chr5H:516,564,134:T: C	Receptor-like protein kinase	5 H	G%2d	−	−7.02	%
HORVU.MOREX.r3.5HG0533710.1	chr5H:578,375,767:G: C	Dehydrogenase/reductase	5 H	RDW	−	−0.005	gm
HORVU.MOREX.r3.6HG0633710.1	chr6H:560,514,671:C: G	LysM receptor-like kinase	6 H	G%10d	−	−11.99	%
HORVU.MOREX.r3.6HG0541280.1	chr6H:7,962,039:G: A	Mitochondrial transcription termination factor-like	6 H	G%2d	−	−2.73	%
HORVU.MOREX.r3.6HG0610180.1	chr6H:480,310,319:G: C	DUF506 family protein	6 H	G%6d	−	−14.16	%
HORVU.MOREX.r3.7HG0744530.1	chr7H:613,938,048:G: A	disease resistance protein (TIR-NBS-LRR class)	7 H	RDW	−	−0.005	gm
HORVU.MOREX.r3.7HG0748160.1	chr7H:620,907,100:C: T	receptor kinase 1	7 H	G%6d	−	−4.39	%
HORVU.MOREX.r3.7HG0641760.1	chr7H:13,838,812:T: A	K + uptake permease 9	7 H	RDW	−	−0.001	gm

Interestingly, some candidate genes were associated with multiple traits and/or identified through different calculation methods. For instance, two highly significant QTNs were detected within the genes *HORVU.MOREX.r3.4HG0395010.1* and *HORVU.MOREX.r3.5HG0423130.1* on chromosomes 4 H and 5 H, respectively. Both of them exhibited a positive effect on germination traits such as G%6d, G%8d, G%10d and GSpeed under control and drought conditions, respectively. The *HORVU.MOREX.r3.4HG0395010.1* was annotated as Nuclear pore complex protein NUP133 and the *HORVU.MOREX.r3.5HG0423130.1* gene annotated as Thaumatin-like protein.

Under control conditions, none of the genes was positively affecting the seedling traits except for SL, which was influenced by *HORVU.MOREX.r3.1HG0064790.1* and *HORVU.MOREX.r3.5HG0430860.1*, which increases the SL by 1.3 and 1.03 cm, respectively. While under drought conditions, RL and RDW were positively affected by three different genes for each trait. The RL was positively increased under drought stress by *HORVU.MOREX.r3.1HG0082530.1*, *HORVU.MOREX.r3.3HG0234440.1* and *HORVU.MOREX.r3.3HG0231420.1* with 0.75, 1.89, and 1.04 cm, respectively. Moreover, an increase of the RDW of 0.05, 0.007 and 0.001 gm were reported for *HORVU.MOREX.r3.2HG0178520.1*, *HORVU.MOREX.r3.5HG0481010.1* and *HORVU.MOREX.r3.7HG0663860.1*, respectively.

## Discussion

Our study aims to unravel the genetic basis of germination and seedling growth traits, which represent critical phases in plant development, particularly under drought-stress conditions. To simulate drought, we applied a 20% PEG_6000_ solution, providing a consistent and controlled stress environment. We utilized a highly diverse global collection of spring barley from the IPK Genebank in Gatersleben, Germany. By focusing on these early developmental stages, we seek to identify key genetic factors that contribute to drought resilience, offering valuable insights for breeding more stress-tolerant barley varieties.

The analysis of variance (ANOVA) for all studied traits revealed high genetic variation among genotypes. Moreover, the high heritability found for all traits will make the selection of line more tolerant to drought stress possible and productive. All genotypes were germinated under the drought treatment. However, we found a highly significant reduction in the G-speed and seedling-measured traits such as fresh and dry weight, shoot and root length, and shoot-root ratio under drought treatment compared to control. This reduction is expected since germination, leaf growth and shoot and root development are highly sensitive to water availability and can be reduced within a few hours after the drought stress is induced^45–48^. This can be considered as a mechanism to tolerate drought stress, as it is known that small plants tend to improve drought tolerance by excreting less water^49,50^. Our barley global population showed a wide range of drought tolerance in all of the studied traits (Fig. 3, Table S2). The drought tolerance is reflected in reducing the loss of water and increasing the root and shoot dry weight. In response to drought stress, the roots of plants sense the stress signal and change their morphology and structure to help absorb water more efficiently^51^. This was consistent with the results of Samarah (2005)^52^. Also, in another study, Slawin et al. found that using 20% (w/v) PEG-induced drought treatment, equivalent to an osmotic potential level of -1.09 MPa, caused significant variation in germination and seedling development traits^53^. In agreement with their results, a high variability for DTI was reported among the accessions in our study. We recommend prioritizing traits with high variability when selecting accessions for drought-tolerance breeding program. Our correlation analysis revealed insightful relationships among the phenotypic traits under both control and drought conditions, highlighting key traits that can serve as reliable indicators of drought resilience. Notably, there is a consistent positive correlation observed between closely related traits like fresh weight, dry weight and length for roots and shoots, indicating that germination and seedling development are closely coordinated. These results are similar to other barley and PEG-induced studies that showed a positive correlation among the same studied traits^19^.

Abiotic stress, including drought tolerance, is a complex quantitative trait that is influenced by climate conditions, plant developmental stages and stress duration and severity. GWAS has proved to be a promising method for finding SNPs in natural populations as well as significant markers-trait associations^10^. Using multi-method GWAS approaches, as described in our work, is an essential for reliable QTL and candidate gene identification, as they combine diverse statistical models to minimize false positives and negatives. Methods such as MLMM (Multi-Locus Mixed Model) and CMLM (Compressed Mixed Linear Model) enhance detection by efficiently accounting for population structure and kinship^41^. FarmCPU (Fixed and Random Model Circulating Probability Unification) further improves QTL detection by separating fixed and random effects, reducing false positives^42^. Additionally, BLINK (Bayesian-information and Linkage-disequilibrium Iteratively Nested Keyway) increases computational efficiency and power by focusing on markers in linkage disequilibrium^43^. Combining these approaches allows for the discovery of both major and minor QTLs, improving precision in candidate gene selection for marker-assisted breeding and gene editing in wheat, a complex polyploid crop. Our results identified a total of 79 significant SNPs distributed across all seven chromosomes. Notably, Chromosome 5 H accounted for the highest proportion, with 21 SNPs (28.4%), followed by 12 SNPs on Chromosome 2 H, 11 SNPs on Chromosome 3 H, and 9 SNPs on Chromosome 1 H. The remaining chromosomes, 4 H, 6 H, and 7 H, each harbored 7 SNPs, indicating a widespread genetic variation associated with the traits of interest across the barley genome. A meta-analysis of QTLs associated with abiotic stress tolerance in barley identified chromosome 5 harbored 12 QTLs related to drought^54^. As a result of the presence of QTLs associated with abiotic stresses on a specific chromosome, major genes impacting multiple traits can be found. Incorporating such chromosomal regions into marker-assisted breeding programs can greatly enhance selection efficiency for developing climate resilience.

With the availability of a well-annotated barley genome, the SNPs could be precisely localized. In this experiment, out of the 79 significant SNPs detected with different calculation methods (Table S5), 44 SNPs were found out of the exonic region, while35 SNPs were found in exonic regions. The most interesting genes from the first group of genes (44 genes) are Sugar transporter protein, K^+^ uptake permease 9, StAR-related lipid transfer protein, WRKY transcription factor, Beta-amylase and others. All these genes are considered abiotic stress regulators genes^55–59^. Plant biochemical and physiological processes such as photosynthesis, enzyme activation, protein synthesis, stomatal movement and osmoregulation which play an important role in response to drought stress, are dependent on sufficient cellular potassium content, which increases proline production and accounts for ca. 2.0–10.0% of the plant dry weight, while at the same time reducing ROS and NADPH toxic activity and increasing water uptake^60^.

The second group, comprising 35 genes containing exonic SNPs, was identified as a set of potential candidate genes in this study. This group of genes included eight kinase protein family genes, especially the Leucine Rich Receptor (LRR) kinase gene on chr 2 H (three genes), 5 H (two genes), 4 H, 6 H and 7 H (one gene/each) were commonly identified in the control and drought stress treatments and associated with RDW, SL and G% 4d, 6d and 10d. These results are agreed with^53^. This protein family has been reported to be involved in seed germination, ABA and ROS signaling in response to drought stress^61,62^. Recent studies have demonstrated that F-box domain proteins, WD40 repeat proteins, and Metacaspase are involved in many biological processes, including cell cycle regulation, apoptosis, autophagy, gene transcription, signal transduction, histone modification, and chromatin assembly under abiotic stress conditions^63^. Based on previous studies, the identification of these genes in our analysis under drought stress conditions, particularly in association with root dry weight (RDW), root length (RL), shoot length (SL), and germination speed (Gspeed), is strongly supported.

In order to gain insight into the natural variation of worldwide barley genotype collection to drought stress, our study employed a 20% PEG treatment at the germination and seedling stage. Some of the tested genotypes demonstrated resilience to the drought stress treatment, exhibiting 100% germination and significantly higher amounts of RDW, RL and SL compared to the other genotypes. These germplasm will form the basis future research in this field, with the aim of testing them their tolerance to drought stress and potentially utilizing them in drought tolerance breeding programs. The genetic regions identified by GWAS and the exonic SNPs that allowed gene selection of candidate genes regulating germination and seedling traits under control and drought stress treatments will serve as the primary source to continue this genetic work by gene expression and/or CRISPR/Cas. The identified loci may be employed as marker-assisted selection tools to strengthen barley drought stress improvement breeding programs.

## Electronic supplementary material

Below is the link to the electronic supplementary material.

Supplementary Material 1

Supplementary Material 2

Supplementary Material 3

Supplementary Material 4

Supplementary Material 5

Supplementary Material 6

Supplementary Material 7

Supplementary Material 8

Supplementary Material 9

## Data Availability

Data will be made available by the corresponding authors: Prof. Dr. A Badr abadr@science.helwan.edu.eg, and Dr. Helmy Youssef youssef@ipk-gatersleben.de on request.
