# Supplementary figures and images for: GWAS identifies novel loci linked to seedling growth traits in highly diverse barley population under drought stress
Source: Sci Rep. 2025 Mar 24;15:10085. doi: 10.1038/s41598-025-94175-y (PMC11933270; doi:10.1038/s41598-025-94175-y)

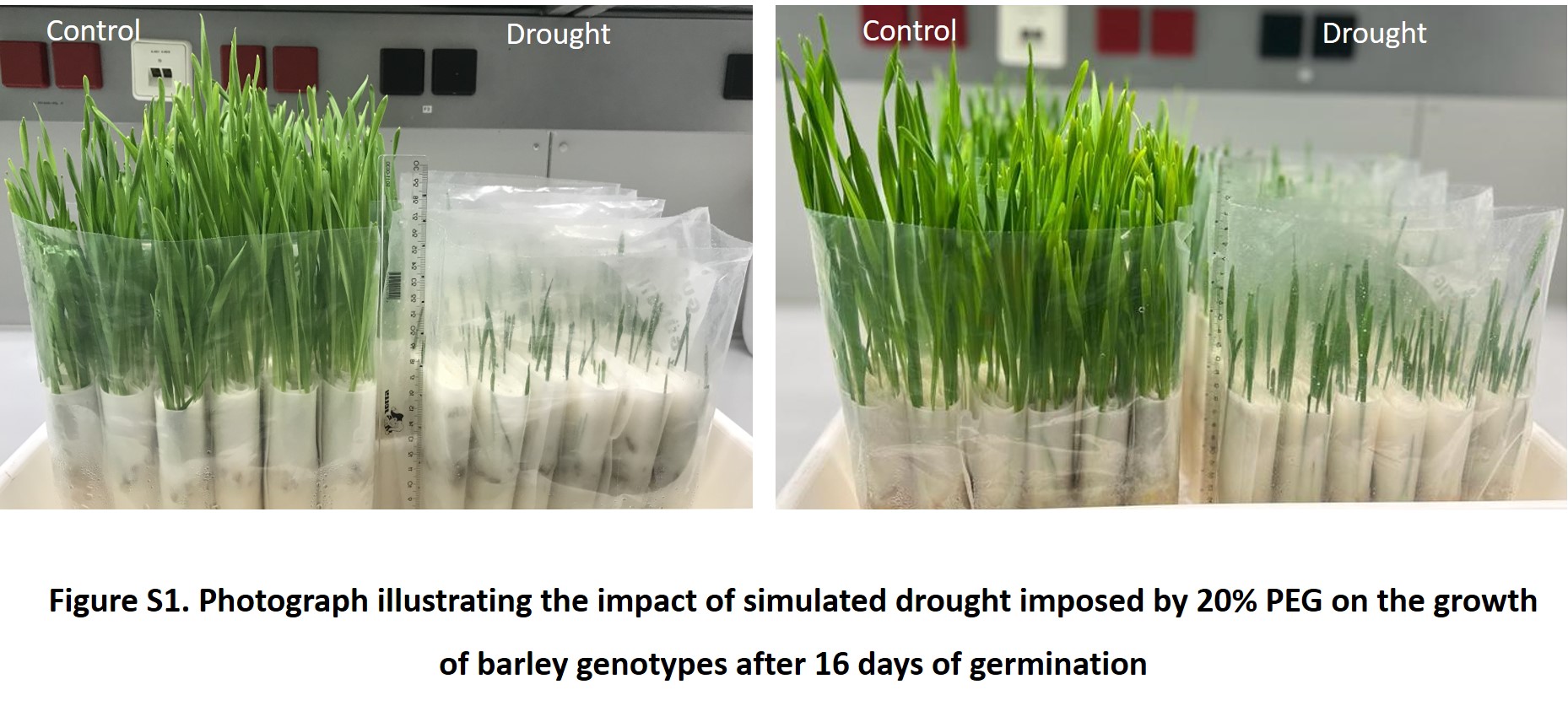

Supplement: Supplementary file 5 — Supplementary Material 5 [file 41598_2025_94175_MOESM5_ESM.jpg]

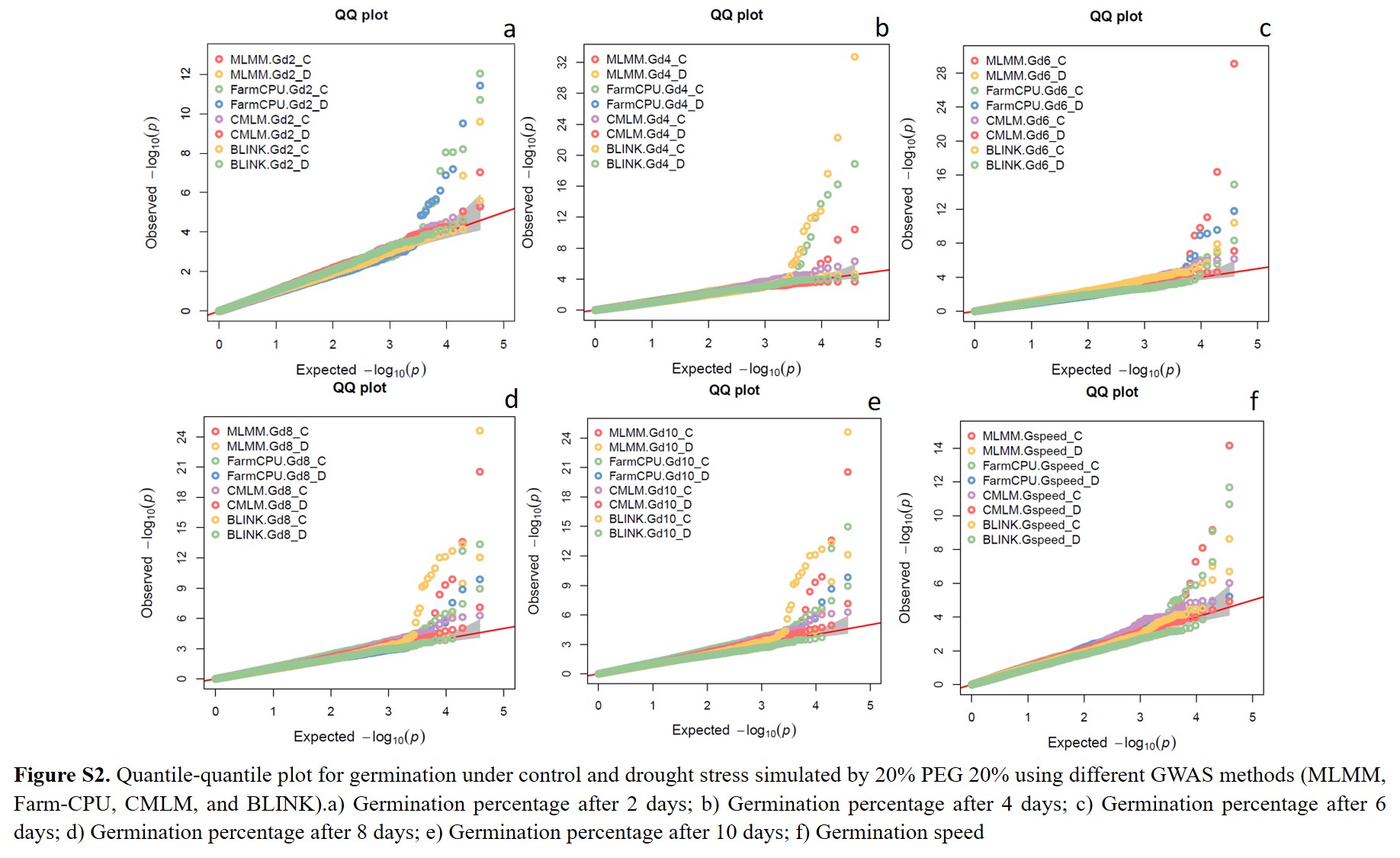

Supplement: Supplementary file 6 — Supplementary Material 6 [file 41598_2025_94175_MOESM6_ESM.jpg]

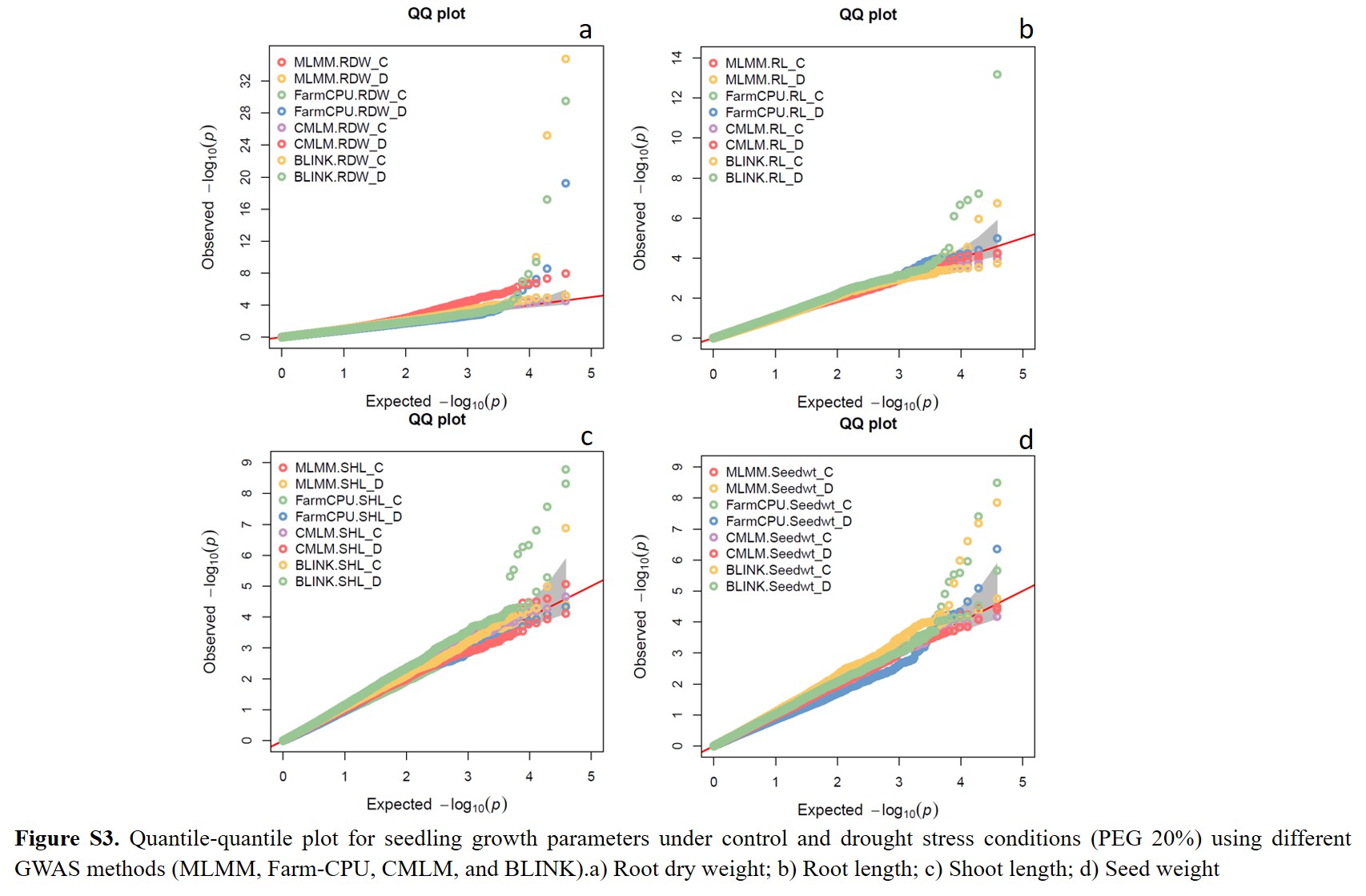

Supplement: Supplementary file 7 — Supplementary Material 7 [file 41598_2025_94175_MOESM7_ESM.jpg]

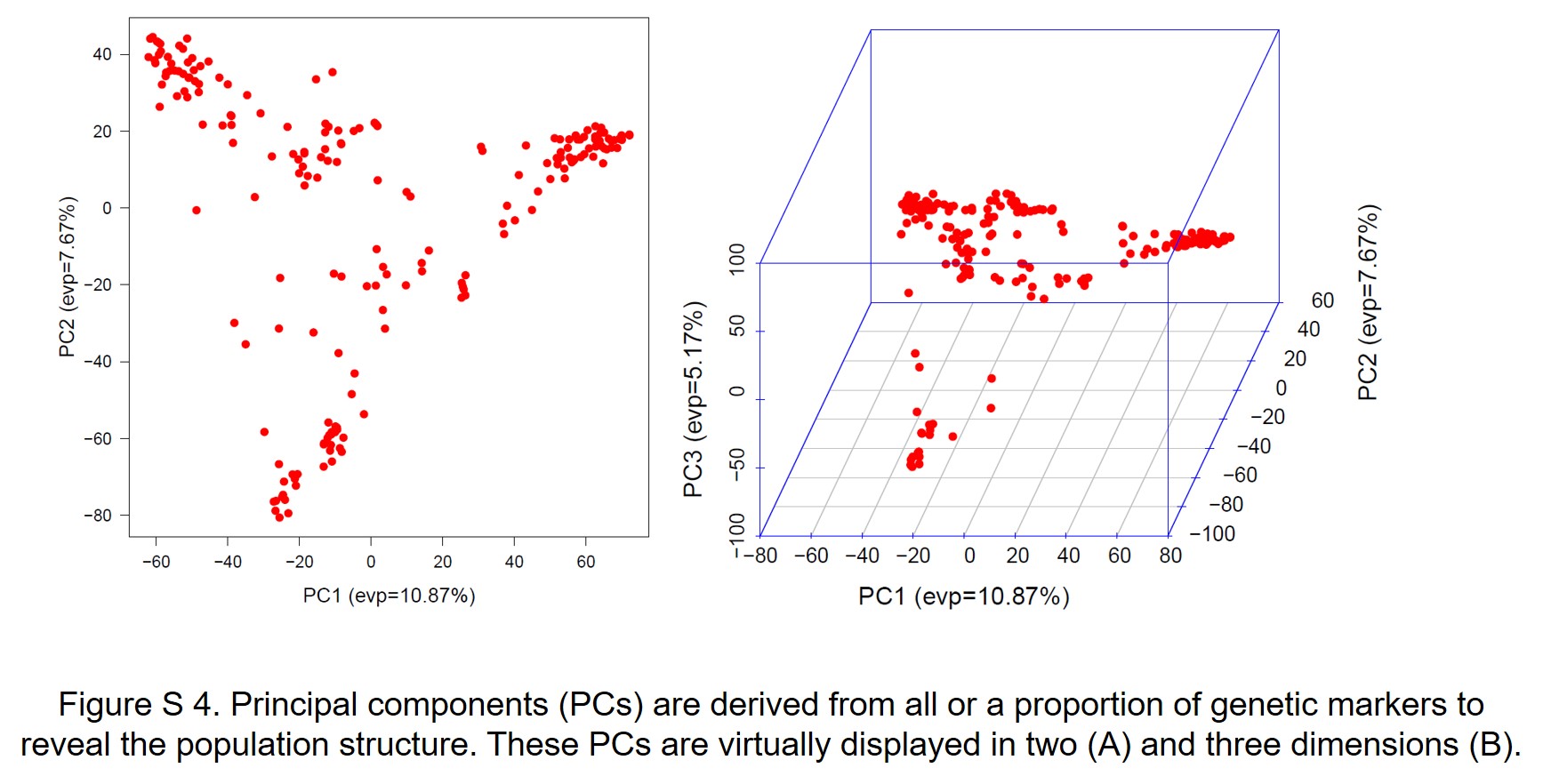

Supplement: Supplementary file 8 — Supplementary Material 8 [file 41598_2025_94175_MOESM8_ESM.jpg]
